# Supplementary material for: Effects of Intestinal Bacterial Hydrogen Gas Production on Muscle Recovery following Intense Exercise in Adult Men: A Pilot Study
Source: Nutrients. 2022 Nov 18;14(22):4875. doi: 10.3390/nu14224875 (PMC9693815; doi:10.3390/nu14224875)
Supplement: Supplementary file 1 [file nutrients-14-04875-s001.zip › nutrients-1901137-supplementary.pdf]

Supplementary Materials

Effects of intestinal bacterial hydrogen gas production on muscle recovery after intense exercise in adult men: A pilot study

Nobuhiko Eda[1,2,†], Saki Tsuno[3,†], Nobuhiro Nakamura[4], Ryota Sone[5], Takao Akama[4], Mitsuharu Matsumoto[3,*]

[1] Waseda Institute for Sport Sciences, Waseda University, Tokorozawa 359-1192, Japan

[2] Department of Fundamental Education, Dokkyo Medical University, Shimotsuga-gun 321-0293, Japan

[3] Dairy Science and Technology Institute, Kyodo Milk Industry Co. Ltd., Hinode-machi, Nishitama-gun 190-0182, Japan

[4] Faculty of Sport Sciences, Waseda University, Tokorozawa 359-1192, Japan

[5] Japan Institute of Sports Sciences, Kita-ku, Tokyo 115-0056, Japan

*Correspondence: m-matumoto@meito.co.jp

Supplementary Methods

Preparation of the test beverage

H_2-producing milk was prepared by adding galactooligosaccharide (2 g), maltitol (2 g), and glucomannan (0.2 g), which are the active ingredients that produce intestinal H_2, to a milk solution comprising whole milk (50%) and skim milk (50%). The lactose (approximately 10 g/200 ml) in the milk was also considered an active ingredient. A beverage without galactooligosaccharide, maltitol, glucomannan, and lactose that had a similar texture and flavor as the H_2-producing milk was used as the placebo. This placebo was made using sucrose, powdered syrup, milk protein, dextrin, unsalted butter, and milk flavor. After sterilization, both beverages were packed into an aluminum pouch. In the study, 200 mL of each beverage was provided in an unlabeled cup.

Determination of 8-hydroxy-2'-deoxyguanosine (8-OHdG)

Whole urine samples were collected before, immediately after, and 60 min after exercise. After measuring the total volume, samples were centrifuged at 2000 × g for 15 min. The 8-hydroxy-2'-deoxyguanosine (8-OHdG) concentration was measured using an enzyme-linked immunosorbent assay kit (New 8-OHdG check; Japan Institute for the Control of Aging, Shizuoka, Japan). A standard curve for 8-OHdG was created by plotting the relation of standard 8-OHdG concentrations and absorbance (450 nm) on a semilogarithmic paper. The 8-OHdG concentration was calculated by each absorbance of the sample and the standard curve. In the present study, 8-OHdG concentration was compensated by the urinary creatinine concentration. The 8-OHdG production rate was calculated as follows: (8-OHdG concentration × total volume of urine sample) / (period elapsed between the last and current collection × body mass).

Extraction of bacterial DNA from the colonic content

Extraction of fecal bacteria DNA was performed according to the Quality Protocol Standard Operating Procedures for Fecal Samples DNA Extraction, Protocol Q, provided by the International Human Microbiome Standards [42]. Approximately 150 to 200 mg of each frozen fecal sample was homogenized with 1 mL of ASL lysis buffer from the Qiagen QIAamp DNA stool kit (QIAGEN Canada, Mississauga, ON, Canada) and 0.3 g of sterile zirconia beads (diameter, 0.1 mm) by vortexing for 2 min and incubating for 15 min at 95°C. The suspension was mechanically homogenized for 60 s at 4,000 rpm using a Micro Smash MS-100 homogenization system (Tomy, Tokyo, Japan) and incubated on ice for 5 min; this was repeated up to eight times. Then, each supernatant was collected by centrifugation (16,000 × g, 5 min, 4°C). The aforementioned procedure was repeated twice. In total, 850 μL of supernatant was collected. Then, 260 μL of 10 M ammonium acetate was added to each supernatant; this combination was mixed well, incubated on ice for 5 min, and centrifuged (16,000 × g, 10 min, 4°C). Then, one volume of isopropanol was added to the supernatant; this combination was mixed well, incubated for 30 min, and centrifuged (16,000 × g, 15 min,

4°C). The supernatant was removed and each pellet was washed with 100 μL of 70% ethanol, dried under a vacuum for 3 min, and dissolved in Tris-EDTA buffer (Nacalai Tesque, Kyoto, Japan.). Finally, DNA was purified using the Qiagen QIAamp DNA stool kit and stored at -80°C.

Construction of the 16S rRNA gene amplicon library and next-generation sequencing

The V1-V2 region of the bacterial 16S rRNA gene was amplified by a polymerase chain reaction (PCR) with fusion primers using fecal DNA as a template. The forward primer contained an Ion A adapter sequence, followed by a key, barcode, adapter (GT), and 27Fmod primer sequence (3′-AGRGTTTGATYMTGGCTCAG-5′) [43]. The reverse primer contained an Ion truncated P1 adapter and 338 R primer sequence (3′-TGCTGCCTCCCGTAGGAGT-5′) [2]. PCR, DNA purification, emulsion PCR, and sequencing were performed using the Ion PGM system (Thermo Fisher Scientific, Waltham, MA, USA) in accordance with the manufacturer's instrAuctions.

Data processing and sequence alignment

Sequence data were obtained in the FASTQ format and analyzed using QIIME software [44]. Raw sequences were sorted according to the respective barcodes and then screened for quality (average quality score ≥20) and primer sequence correctness. Trimmed sequences were clustered into operational taxonomic units at a level of 97%, similar to the UCLUST method [45] and the farthest neighbor algorithm. The most abundant sequence in each operational taxonomic unit was chosen as the representative sequence and aligned with the Python nearest alignment space termination (PyNAST) algorithm [46], which is the default alignment method of QIIME, against the Greengenes core set [47]. Potentially chimeric sequences were identified and removed by the ChimeraSlayer algorithm. Non-chimeric representative sequences were assigned to taxons using the RDP classifier with a confidence cut-off value of 80% [48].

REFERENCES

42. Costea, P.I.; Zeller, G.; Sunagawa, S.; Pelletier, E.; Alberti, A.; Levenez, F.; Tramontano, M.; Driessen, M.; Hercog, R.; Jung, F.E., et al. Towards standards for human fecal sample processing in metagenomic studies. *Nat Biotechnol* **2017**, *35*, 1069-1076, doi:10.1038/nbt.3960.
43. Kim, S.W.; Suda, W.; Kim, S.; Oshima, K.; Fukuda, S.; Ohno, H.; Morita, H.; Hattori, M. Robustness of gut microbiota of healthy adults in response to probiotic intervention revealed by high-throughput pyrosequencing. *DNA Res* **2013**, *20*, 241-253, doi:10.1093/dnares/dst006.
44. Caporaso, J.G.; Kuczynski, J.; Stombaugh, J.; Bittinger, K.; Bushman, F.D.; Costello, E.K.; Fierer, N.; Pena, A.G.; Goodrich, J.K.; Gordon, J.I., et al. QIIME allows analysis of high-throughput community sequencing data. *Nat Methods* **2010**, *7*, 335-336, doi:10.1038/nmeth.f.303.
45. Edgar, R.C. Search and clustering orders of magnitude faster than BLAST. *Bioinformatics* **2010**, *26*, 2460-2461, doi:10.1093/bioinformatics/btq461.
46. Caporaso, J.G.; Bittinger, K.; Bushman, F.D.; DeSantis, T.Z.; Andersen, G.L.; Knight, R. PyNAST: a flexible tool for aligning sequences to a template alignment. *Bioinformatics* **2010**, *26*, 266-267, doi:10.1093/bioinformatics/btp636.

47. DeSantis, T.Z.; Hugenholtz, P.; Larsen, N.; Rojas, M.; Brodie, E.L.; Keller, K.; Huber, T.; Dalevi, D.; Hu, P.; Andersen, G.L. Greengenes, a chimera-checked 16S rRNA gene database and workbench compatible with ARB. *Appl Environ Microbiol* **2006**, *72*, 5069-5072, doi:10.1128/AEM.03006-05.
48. Wang, Q.; Garrity, G.M.; Tiedje, J.M.; Cole, J.R. Naive Bayesian classifier for rapid assignment of rRNA sequences into the new bacterial taxonomy. *Appl Environ Microbiol* **2007**, *73*, 5261-5267, doi:10.1128/aem.00062-07.

Supplementary Table S1. The measurement values of serum and urine during trial

		Pre	Post 0	Post 30	Post 60	Next morning	Parametric analyses *p* value			Non-parametric analyses	
							Interaction (η_p^2)	Time (η_p^2)	Trial (η_p^2)	χ^2	*p* value
Serum											
Lactate (mg/dL)[a]	HPM	7.6 ± 2.2	22.1 ± 5.3 ††	10.6 ± 4.1	8.2 ± 2.1	7.8 ± 3.8	0.108	< 0.001 (0.781)	0.308		
	PLA	6.7 ± 2.4	28.9 ± 9.7 ††	10.5 ± 3.8	9.3 ± 2.4	8.2 ± 2.6	(0.285)	< 0.001 (0.829)	(0.147)		
LPO (nmol/mL)[a]	HPM	3.0 ± 0.5	3.1 ± 0.3	3.3 ± 0.5	3.4 ± 0.5	3.4 ± 0.6	0.584	0.105 (0.232)	0.933		
	PLA	3.1 ± 0.5	3.2 ± 0.4	3.5 ± 0.6	3.4 ± 0.5	3.2 ± 0.4	(0.094)	0.102 (0.234)	(0.001)		
UA (mg/dL)[a]	HPM	5.7 ± 0.6	6.0 ± 0.8	6.9 ± 0.9 ††	6.9 ± 1.0 ††	7.2 ± 0.7 ††	0.248	< 0.001 (0.811)	0.633		
	PLA	5.5 ± 0.7	5.9 ± 0.8	6.7 ± 1.0 ††	6.6 ± 0.9 ††	7.2 ± 0.8 ††	(0.170)	< 0.001 (0.766)	(0.034)		
Creatine kinase (U/L)[b]	HPM	124.5 (46.5)	132.8 (52.8)	141.5 (68.4)†	151.9 (88.5)†	161.3 (267.4)†				13.2	0.01
	PLA	141.0 (40.8)	141.4 (44.2)	143.3 (57.2)†	141.6 (92.5)	158.7 (162.8)†				12.2	0.016
Myoglobin (ng/mL)[b]	HPM	20.1 (5.6)	25.9 (21.1)	50.6 (20.2)††	56.5 (42.5)††	44.2 (12.1)†				23.2	< 0.001
	PLA	23.4 (2.7)	28.6 (23.5)	52.3 (51.7)†,‡	49.7 (58.7)††,‡‡	47.2 (11.6)				22.7	< 0.001
Leukocyte (/µL)[b]	HPM	4955 (528)	6792 (1161)	15114 (4448)††	15831 (4049)††	5403 (608)¶¶				25.83	< 0.001
	PLA	5430 (483)	8448 (3199)	15778 (4413)††	15418 (4617)††	4988 (808)§§,¶¶				28.9	< 0.001
Urea nitorogen (mg/dL)[b]	HPM	12.2 (3.1)	11.5 (2.6)	13.7 (2.2)‡‡	13.5 (1.6)‡	14.2 (2.3)				17.3	0.002
	PLA	13.2 (3.7)	12.5 (3.0)	13.9 (2.9)‡‡	14.0 (2.1)	14.5 (4.0)‡‡				21.2	< 0.001
Urine											
8-OHdG concentration	HPM	8.0 ± 2.3	9.4 ± 1.5		6.1 ± 1.2		0.175	0.007 (0.558)	0.746		
(ng/mg crea.)[a]	PLA	6.6 ± 1.6	11.0 ± 3.8 ††		6.8 ± 2.0		(0.252)	0.006 (0.577)	(0.019)		
8-OHdG production	HPM	10.7 ± 4.0 *	10.8 ± 2.4		7.1 ± 2.1		0.039	0.077 (0.348)	0.782		
rate (ng/kg/hr)[a]	PLA	6.1 ± 2.6	14.2 ± 5.7 ††		7.6 ± 2.3		(0.418)	0.006 (0.579)	(0.014)		

HPM: H_2-producing milk, PLA: placebo, LPO: lipid peroxide, UA: uric acid, BUN: blood urea nitrogen, Cr: creatinine, 8-OHdG: 8-hydroxy-2'-deoxyguanosine.

Pre: before exercise, Post 0: immediately after exercise, Post 30: 30 min after exercise, Post 60: 60 min after exercise, Next morning: the morning after the trial.

[a]Parametric analyses: Mean ± SD. ††$p < 0.01$ vs Pre, *$p < 0.05$ vs PLA (A two-way repeated measures ANOVA and Dunnett's multiple comparison test).

[b]Non-parametric analyses: Median (interquartile range), †$p < 0.05$, ††$p < 0.01$ vs. Pre, ‡$p < 0.05$, ‡‡$p < 0.01$ vs. Post, §§$p < 0.05$ vs. Post 30, ¶¶$p < 0.01$ vs. Post 60 (Friedman test and adjusted using the Bonferroni method).

Supplementary Table S2. Respiratory variables during exercise session.

		0 min	3 min	15 min	30 min	45 min	60 min	Parametric analyses *p* value			Non-parametric analyses	
								Interaction (η_p^2)	Time (η_p^2)	Trial (η_p^2)	χ^2	*p* value
$\dot{V}O_2$ (mL/min)[a]	HPM	283 ± 60	2287 ± 428**	2409 ± 385**	2310 ± 499**	2098 ± 470**	2171 ± 430**	0.794	< 0.001 (0.944)	0.929		
	PLA	270 ± 57	2342 ± 572**	2395 ± 463**	2298 ± 717**	2074 ± 524**	2141 ± 485**	(0.046)	< 0.001 (0.920)	(0.001)		
$\dot{V}CO_2$ (mL/min)[b]	HPM	234 (65)	1963 (367)	2306 (482)††	2151 (526)††	1861 (599)	1848 (368)				30.2	< 0.001
	PLA	221 (52)	2040 (565)††	2257 (669)††	1926 (943)†	1706 (712)§	1751 (462)				28.07	< 0.001
Heart rate (bpm)[a]	HPM	65.0 ± 8.0	147.3 ± 5.1**	161.3 ± 6.9**	161.4 ± 8.5**	156.5 ± 13.5**	161.4 ± 11.5**	0.375	< 0.001 (0.958)	0.913		
	PLA	61.5 ± 6.9	150.0 ± 10.9**	162.4 ± 7.2**	159.6 ± 12.6**	159.0 ± 11.0**	161.9 ± 9.3**	(0.137)	< 0.001 (0.971)	(0.002)		
$\dot{V}E$ (L/min)[b]	HPM	9.7 (1.5)	72.1 (17.8)††	75.9 (14.6)††	67.3 (22.5)††	58.3 (10.5)	57.2 (5.9)				27.36	< 0.001
	PLA	9.3 (3.2)	72.9 (22.8)††	74.9 (19.4)††	62.6 (39.3)†	56.4 (24.3)	58.3 (18.3)				26.64	< 0.001
$\dot{V}E/\dot{V}CO_2$[a]	HPM	40.6 ± 4.7	37.2 ± 3.6*	32.8 ± 1.9**	32.7 ± 3.0**	32.2 ± 3.5**	32.0 ± 4.3**	0.896	< 0.001 (0.745)	0.248		
	PLA	41.1 ± 4.7	37.7 ± 5.0*	34.1 ± 4.6**	33.5 ± 5.0**	33.9 ± 5.6**	32.6 ± 5.1**	(0.044)	< 0.001 (0.668)	(0.185)		
$\dot{V}E/\dot{V}O_2$[b]	HPM	32.0 (3.8)	31.5 (3.5)	31.2 (0.7)	29.9 (2.3)	27.6 (3.0)††	28.3 (2.2)†				17.86	0.003
	PLA	33.0 (2.7)	34.5 (8.8)	31.6 (8.1)	29.0 (7.0)	28.1 (9.6)	29.3 (5.1)†,‡				16.86	0.005

HPM: H_2-producing milk producing milk, PLA: placebo

[a]Parametric analyses: Mean ± SD. *$p < 0.05$, **$p < 0.01$ vs. Pre (A two-way repeated measures ANOVA and Dunnett's multiple comparison test).

[b]Non-parametric analyses: Median (interquartile range), †p <0.05, ††p < 0.01 vs 0 min, ‡p < 0.05 vs 3 min, §p < 0.05 vs. 15 min (Friedman test and adjusted using the Bonferroni method)

Supplementary Table S3. The value of energy expenditure (kcal/min)

	HPM						PLA						AUC	
	0	3	15	30	45	60	0	3	15	30	45	60	HPM	PLA
MDH01	1.50	12.19	11.75	11.62	10.19	9.79	1.69	13.42	13.16	13.50	11.65	10.39	563.23	634.47
MDH02	1.04	8.03	9.26	7.75	7.44	7.24	1.16	7.85	9.45	7.24	7.65	8.21	406.83	403.81
MDH03	1.56	8.43	10.12	9.57	8.79	9.98	1.21	9.01	9.18	8.80	7.88	8.59	458.96	435.08
MDH04	1.35	10.52	10.48	9.38	7.83	10.08	1.37	9.91	10.31	8.44	8.64	8.87	475.00	456.19
MDH06	0.89	11.07	12.46	11.67	10.84	11.07	0.88	11.29	11.21	9.03	9.59	9.61	620.08	536.15
MDH07	1.60	13.06	14.57	14.87	13.98	14.16	1.09	12.38	14.10	15.10	12.89	13.87	740.16	743.21
MDH08	1.21	13.89	14.05	14.52	13.18	13.11	1.64	17.16	15.58	16.84	14.75	14.81	736.69	828.29
MDH10	1.68	11.54	12.64	11.57	10.15	9.43	1.34	11.19	12.14	11.65	8.64	10.00	555.62	548.81
mean	1.35	11.09	11.92	11.37	10.30	10.61	1.30	11.53	11.89	11.32	10.21	10.54	569.57	573.25
SD	0.28	2.07	1.88	2.47	2.35	2.18	0.27	2.90	2.28	3.51	2.59	2.47	123.91	151.73

HPM: Hydrogen gas producing milk

PLA: Placebo

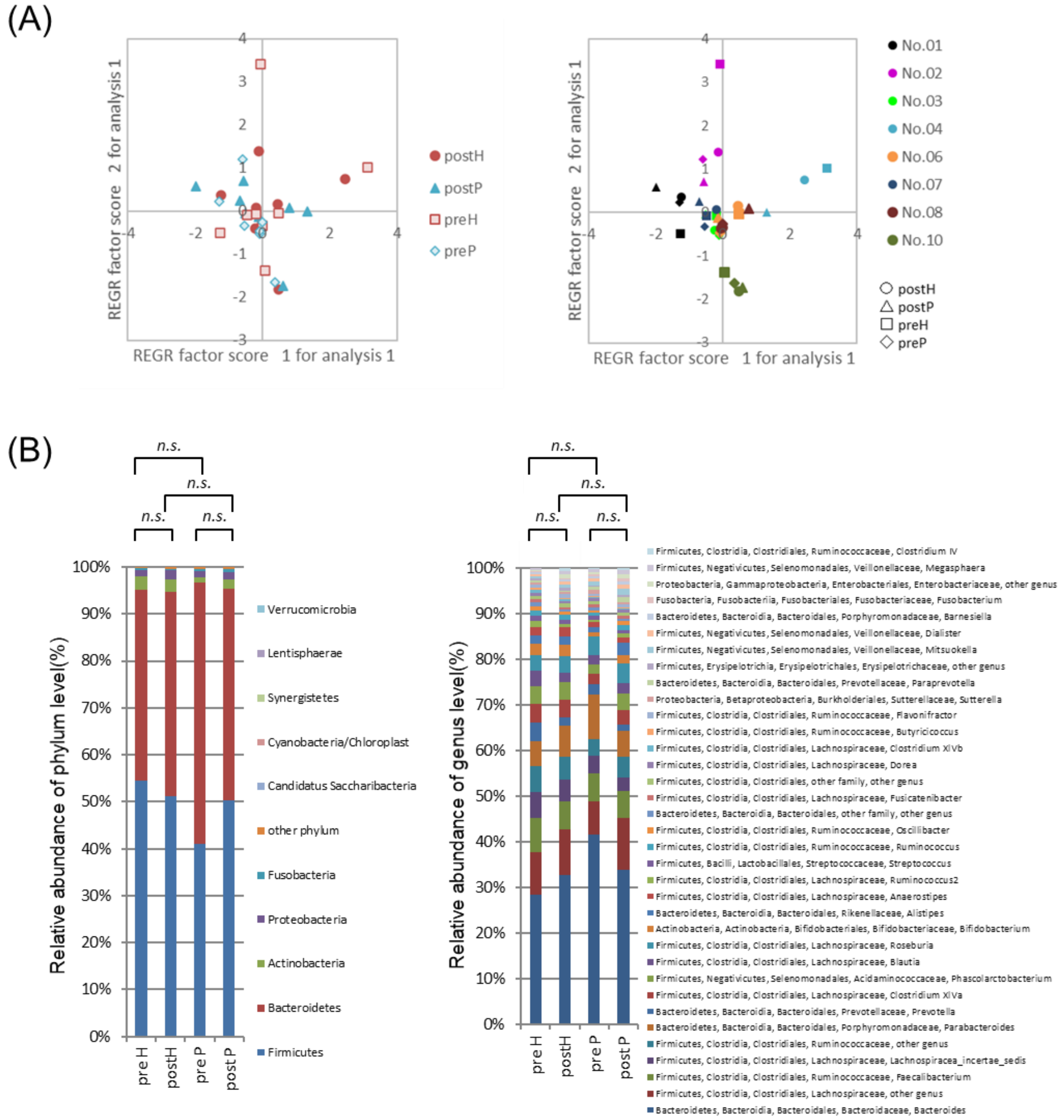

Supplementary Figure S1. Alterations of the fecal microbiota with the consumption of H_2-producing milk and placebo. preH, before exercise during the H_2-producing milk trial; postH, after exercise during the H_2-producing milk trial, preP, before exercise during the placebo trial; postP, after exercise during the placebo trial. (A) Principal component analysis (PCA) of fecal microbiota. A PCA of the fecal microbiota data was performed to evaluate the influence of the consumption of the H_2-producing milk and the consumption of the placebo (left). A PCA of the fecal microbiota data was also performed to evaluate the alterations of the microbiota of the individuals (right). (B) Relative abundance of the fecal microbiota before and after exercise during the H_2-producing milk and placebo trials. Bacterial groups with a relative abundance of less than 0.5% are not shown.

Participant raw data

ID	Age	Height (cm)	Body Mass (kg)	Body Fat (%)	BMI	VO2max/W (ml/kg/min)
MDH01	29	172.4	57.2	13.9	19.25	38.8
MDH02	24	168.1	60.7	16.1	21.48	46.3
MDH03	25	171.2	64.5	15.3	22.01	36.3
MDH04	24	165.6	49.6	6.9	18.09	57.2
MDH05	32	174.3	79.9	19.7	26.30	38.6
MDH06	22	174.3	60.3	11.2	19.85	63.5
MDH07	25	175.0	80.4	22.6	26.25	52.3
MDH08	24	178.0	71.8	17.4	22.66	53.3
MDH09	25	170.7	56.1	8	19.25	69.7
MDH10	32	170.0	64.5	15.5	22.32	49.8

Breath H_2 concentration raw data

	H_2-producing milk						Placebo				
	0h	5h	6h	7h	AUC		0h	5h	6h	7h	AUC
MDH01	10.481	47.947	46.701	35.385	86.138		38.724	3.406	2.587	2.534	19.02
MDH02	5.798	26.19	50.287	56.027	89.9955		7.718	2.407	4.364	3.085	4.9515
MDH03	14.842	60.687	64.48	57.778	116.951		6.525	3.336	3.753	5.748	3.2175
MDH04	7.868	47.458	14.701	17.711	51.3445		3.919	4.53	8.789	8.253	7.648
MDH05	14.381	5.897	7.683	6.942	6.5505		16.507	3.706	3.905	4.535	7.014
MDH06	3.734	22.442	21.519	24.426	46.839		9.606	4.561	4.185	3.64	4.449
MDH07	14.22	34.256	15.183	23.169	25.4735		7.46	2.808	8.979	16.147	15.1665
MDH08	2.061	14.724	29.241	41.259	59.442		44.684	15.196	12.378	15.064	20.314
MDH09	21.078	15.285	13.413	8.823	17.1795		7.511	2.165	4.013	4.089	5.483
MDH10	11.328	30.793	39.576	34.396	59.247		8.978	3.886	4.57	3.247	4.8275

Participants 5 and 9 whose breath H2 concentration AUCs with H_2-producing milk ingestion were smaller than the maximum value of the breath H2 concentration AUC with placebo ingestion.

Respiratory gas raw data (1)

	HPM						PLA					
**VO2**	rest	3 min	15 min	30 min	45 min	60 min	rest	3 min	15 min	30 min	45 min	60 min
MDH01	217	1669	1898	1570	1517	1512	238	1571	1910	1463	1527	1668
MDH02	290	2179	2113	1923	1635	2089	290	2058	2079	1720	1757	1812
MDH03	321	1691	2008	1929	1763	2018	249	1813	1831	1764	1614	1736
MDH04	184	2294	2486	2378	2203	2255	184	2277	2254	1830	1934	1963
MDH05	319	2510	2220	2326	2299	2078	280	2457	2236	2307	2199	2207
MDH06	316	2540	2388	2350	2077	1990	356	2706	2640	2736	2375	2107
MDH07	251	2824	2856	2936	2671	2694	339	3415	3150	3442	2989	2978
MDH08	336	2691	2954	3015	2834	2855	230	2558	2835	3045	2605	2782
MDH09	257	2767	2870	2393	2291	2329	249	2795	2999	2549	2499	2315
MDH10	349	2410	2569	2377	2085	1953	275	2338	2461	2387	1794	2082

	HPM						PLA					
**VO2/W**	rest	3 min	15 min	30 min	45 min	60 min	rest	3 min	15 min	30 min	45 min	60 min
MDH01	3.8	29.2	33.2	27.5	26.5	26.4	4.2	27.5	33.4	25.6	26.7	29.2
MDH02	4.8	35.9	34.8	31.7	26.9	34.4	4.8	33.9	34.2	28.3	28.9	29.9
MDH03	5	26.2	31.1	29.9	27.3	31.3	3.9	26.6	28.4	27.4	25	26.9
MDH04	3.7	46.2	50.1	47.9	44.4	45.5	3.7	45.9	45.4	36.9	39	39.6
MDH05	4	31.4	27.8	29.1	28.8	26	3.5	30.8	28	28.9	27.5	27.6
MDH06	5.2	42.1	39.6	39	34.4	33	5.9	44.9	43.8	45.4	39.4	34.9
MDH07	3.1	35.1	35.5	36.5	33.2	33.5	4.2	42.5	39.2	42.8	37.2	37
MDH08	4.7	37.5	41.1	42	39.5	39.8	3.2	35.6	39.5	42.4	36.3	38.7
MDH09	4.6	49.3	51.2	42.7	40.8	41.5	4.4	49.8	53.5	45.4	44.5	41.3
MDH10	5.4	37.4	39.8	36.8	32.3	30.3	4.3	36.2	38.2	37	27.8	32.3

	HPM						PLA					
**VCO2**	rest	3 min	15 min	30 min	45 min	60 min	rest	3 min	15 min	30 min	45 min	60 min
MDH01	178	1402	1686	1430	1456	1376	207	1540	1852	1405	1512	1552
MDH02	224	1852	2004	1708	1304	1784	223	1729	1979	1560	1617	1676
MDH03	277	1640	2037	1825	1564	1894	219	1722	1813	1712	1437	1637
MDH04	164	1950	2473	2156	2020	2053	147	2163	2168	1709	1794	1776
MDH05	272	2083	2152	2144	2246	1881	221	1892	2150	2114	1969	1869
MDH06	213	2108	2205	2208	1889	1802	285	2571	2572	2519	2150	1935
MDH07	243	2598	2625	2664	2482	2361	288	3415	2953	3111	2835	2819
MDH08	267	2341	2815	2815	2626	2675	182	2200	2747	2903	2432	2709
MDH09	215	2463	2863	2239	2060	2163	197	2460	2944	2384	2273	2046
MDH10	288	1976	2407	2145	1832	1698	242	1917	2346	2140	1498	1725

	HPM						PLA					
**VE**	rest	3 min	15 min	30 min	45 min	60 min	rest	3 min	15 min	30 min	45 min	60 min
MDH01	8.2	56.1	60.1	54.8	57.2	57.1	10.3	64.2	73.5	55.5	61.6	65.2
MDH02	9.7	70.0	68.0	55.2	42.2	56.2	9.7	71.0	67.0	49.5	51.1	52.3
MDH03	9.1	59.4	62.1	54.5	44.7	51.6	7.6	52.7	53.1	48.4	41.8	45.9
MDH04	6.1	66.2	78.5	61.8	59.3	57.9	6.1	78.4	64.1	49.1	50.0	48.0
MDH05	9.4	67.5	58.9	62.0	67.2	54.5	8.2	63.0	60.3	60.0	57.4	54.1
MDH06	9.7	93.2	73.2	74.1	59.5	57.2	11.7	118.9	97.6	101.7	84.1	62.6
MDH07	10.6	86.6	89.1	89.8	87.0	75.3	12.5	126.3	118.0	116.4	117.2	108.6
MDH08	10.3	80.9	84.0	88.0	79.1	81.9	7.0	74.7	81.7	84.4	70.5	82.6
MDH09	8.0	76.7	95.9	69.9	65.1	67.6	7.3	79.8	100.5	75.3	73.2	65.5
MDH10	10.8	74.2	80.1	72.8	57.0	56.4	8.8	66.2	76.2	69.6	48.6	53.9

	HPM						PLA					
**VE/VO2**	rest	3 min	15 min	30 min	45 min	60 min	rest	3 min	15 min	30 min	45 min	60 min
MDH01	37.788	33.613	31.665	34.904	37.706	37.765	43.277	40.866	38.482	37.936	40.341	39.089
MDH02	33.448	32.125	32.182	28.705	25.810	26.903	33.448	34.500	32.227	28.779	29.084	28.863
MDH03	28.349	35.127	30.926	28.253	25.355	25.570	30.522	29.068	29.001	27.438	25.898	26.440
MDH04	33.152	28.858	31.577	25.988	26.918	25.676	33.152	34.431	28.438	26.831	25.853	24.452
MDH05	29.467	26.892	26.532	26.655	29.230	26.227	29.286	25.641	26.968	26.008	26.103	24.513
MDH06	30.696	36.693	30.653	31.532	28.647	28.744	32.865	43.939	36.970	37.171	35.411	29.710
MDH07	42.231	30.666	31.197	30.586	32.572	27.951	36.873	36.984	37.460	33.818	39.210	36.467
MDH08	30.655	30.063	28.436	29.187	27.911	28.687	30.435	29.203	28.818	27.718	27.063	29.691
MDH09	31.128	27.720	33.415	29.210	28.416	29.025	29.317	28.551	33.511	29.541	29.292	28.294
MDH10	30.946	30.788	31.179	30.627	27.338	28.879	32.000	28.315	30.963	29.158	27.090	25.889

	HPM						PLA					
**VE/VCO2**	rest	3 min	15 min	30 min	45 min	60 min	rest	3 min	15 min	30 min	45 min	60 min
MDH01	46.1	40.0	35.6	38.3	39.3	41.5	49.7	41.7	39.7	39.5	40.7	42.0
MDH02	43.3	37.8	33.9	32.3	32.4	31.5	43.4	41.1	33.9	31.7	31.6	31.2
MDH03	32.9	36.2	30.5	29.9	28.6	27.2	34.7	30.6	29.3	28.3	29.1	28.0
MDH04	37.2	34.0	31.7	28.7	29.4	28.2	41.4	36.2	29.6	28.7	27.9	27.0
MDH05	34.6	32.4	27.4	28.9	29.9	29.0	37.1	33.3	28.0	28.4	29.2	28.9
MDH06	45.5	44.2	33.2	33.6	31.5	31.7	41.1	46.3	37.9	40.4	39.1	32.4
MDH07	43.6	33.3	33.9	33.7	35.1	31.9	43.4	37.0	40.0	37.4	41.3	38.5
MDH08	38.6	34.6	29.8	31.3	30.1	30.6	38.5	34.0	29.7	29.1	29.0	30.5
MDH09	37.2	31.1	33.5	31.2	31.6	31.3	37.1	32.4	34.1	31.6	32.2	32.0
MDH10	37.5	37.5	33.3	33.9	31.1	33.2	36.4	34.5	32.5	32.5	32.4	31.2

Respiratory gas raw data (2)

	HPM						PLA					
**HR**	rest	3 min	15 min	30 min	45 min	60 min	rest	3 min	15 min	30 min	45 min	60 min
MDH01	74	157	160	154	150	150	66	164	170	156	162	163
MDH02	70	140	157	148	133	147	66	149	156	154	158	158
MDH03	71	143	160	163	153	161	61	127	150	145	141	152
MDH04	54	149	168	156	153	156	52	154	159	141	150	152
MDH05	71	159	170	170	177	174	70	155	166	176	171	172
MDH06	65	150	150	167	161	163	62	158	169	170	168	168
MDH07	66	147	157	160	166	168	58	151	161	167	166	169
MDH08	52	147	168	173	180	184	54	145	169	176	175	178
MDH09	60	159	177	165	155	156	69	153	172	155	159	146
MDH10	68	145	170	170	156	162	73	152	165	168	152	155

	HPM					PLA				
**RPE**	3 min	15 min	30 min	45 min	60 min	3 min	15 min	30 min	45 min	60 min
MDH01	15	16	18	19	20	14	17	18	16	15
MDH02	14	15	13	13	14	13	14	13	13	14
MDH03	13	16	15	14	14	12	15	15	13	13
MDH04	15	16	15	15	15	17	16	14	15	16
MDH05	16	17	18	19	19	15	17	17	19	19
MDH06	15	17	19	19	18	15	17	19	19	18
MDH07	14	15	16	16	17	16	17	18	19	19
MDH08	15	16	16	16	17	13	15	16	16	16
MDH09	14	16	15	14	14	13	17	16	19	20
MDH10	13	17	18	19	18	13	16	18	12	14

	HPM						PLA					
**Respiratory exchange ratio**	rest	3 min	15 min	30 min	45 min	60 min	rest	3 min	15 min	30 min	45 min	60 min
MDH01	0.81	0.84	0.89	0.93	0.91	0.82	0.87	0.98	0.94	0.94	0.99	0.92
MDH02	0.73	0.85	0.95	0.89	0.82	0.85	0.77	0.84	0.95	0.91	0.92	0.9
MDH03	0.87	0.97	1.01	0.95	0.97	0.94	0.88	0.95	0.99	0.97	0.89	0.94
MDH04	0.84	0.85	0.99	0.91	0.92	0.91	0.8	0.95	0.96	0.93	0.95	0.9
MDH05	0.83	0.83	0.97	0.92	0.94	0.89	0.79	0.77	0.92	0.91	0.88	0.84
MDH06	0.78	0.83	0.92	0.94	0.91	0.92	0.8	0.95	0.97	0.93	0.91	0.93
MDH07	0.86	0.92	0.92	0.94	0.93	0.88	0.85	1	0.94	0.9	0.93	0.96
MDH08	0.8	0.87	0.93	0.93	0.93	0.95	0.79	0.86	0.96	0.95	0.94	0.97
MDH09	0.83	0.89	1	0.94	0.9	0.92	0.79	0.88	0.96	0.94	0.88	0.88
MDH10	0.83	0.82	0.92	0.88	0.88	0.85	0.88	0.82	0.93	0.89	0.84	0.83

	HPM						PLA					
**Lipid oxidation**	rest	3 min	15 min	30 min	45 min	60 min	rest	3 min	15 min	30 min	45 min	60 min
MDH01	93	399	359	174	219	378	53	38	193	139	17	214
MDH02	183	422	152	363	488	515	123	427	168	270	236	310
MDH03	74	64	0	152	97	209	69	115	21	89	300	168
MDH04	124	492	51	374	310	342	122	170	157	204	174	316
MDH05	93	539	119	286	213	377	144	717	298	309	454	591
MDH06	70	586	309	239	294	244	118	162	115	309	380	238
MDH07	96	323	391	285	319	492	86	0	297	560	353	220
MDH08	116	467	353	339	351	234	82	554	182	240	258	123
MDH09	78	410	11	260	391	285	160	463	188	279	481	463
MDH10	102	613	362	483	427	470	58	639	273	443	500	604

	HPM						PLA					
**Carbohydrate oxidation**	rest	3 min	15 min	30 min	45 min	60 min	rest	3 min	15 min	30 min	45 min	60 min
MDH01	275	1657	2988	2715	2688	1302	333	3243	3861	2925	3691	2828
MDH02	97	2089	4269	3030	1559	2671	187	1941	4428	3397	3258	2887
MDH03	551	3529	5360	3889	3743	4061	499	3140	4270	4034	2569	3557
MDH04	569	2336	5495	4130	4017	3983	301	4092	4808	3530	3962	3374
MDH05	355	2115	4709	4247	4234	3139	306	1074	4148	3767	3172	2427
MDH06	121	2158	4492	4755	3674	3294	299	4502	6131	5293	4094	3994
MDH07	511	4219	5260	6000	5160	3796	422	6415	6429	5886	5582	6274
MDH08	258	3161	5472	5935	5410	5741	166	3100	6167	6516	5047	6452
MDH09	292	3640	7183	4756	3823	4116	325	3662	6589	5054	3715	3495
MDH10	363	2087	4643	3565	3114	2307	404	1953	4846	3638	1997	2195

Blood raw data (1)

	HPM					PLA				
**LPO**	Pre	Post 0	Post 30	Post 60	Next morning	Pre	Post 0	Post 30	Post 60	Next morning
MDH01	2.5	3.654932	4.172912	4.305375	4.253024	3.1	3.724386	4.186331	4.179781	2.969845
MDH02	3.8	3.11151	3.6	3.785193	4.145311	2.9	3.346295	3.201585	3.332939	3.518832
MDH03	2.6	2.819666	2.831169	3.424556	3.003516	3.1	2.82543	3.175939	3.343788	3.681875
MDH04	3.2	3.160937	3.443819	3.341482	3.560052	2.7	2.941075	2.915586	2.947631	2.468428
MDH05	2.9	2.952235	3.168514	2.981154	3.324083	3.6	3.388826	3.355102	3.602943	3.4334
MDH06	2.8	3.171907	2.849788	3.086034	3.427834	3.5	3.343388	3.695886	3.846723	3.780219
MDH07	2.5	2.618819	2.816827	2.944615	2.885081	2.9	3.047141	3.54745	3.376082	3.249421
MDH08	3.3	3.29416	3.430353	2.771268	2.553688	2.5	2.589307	2.55195	2.606365	2.83946
MDH09	2.7	3.135925	3.150172	3.191924	2.257233	3	3.302892	3.394491	3.188764	3.144653
MDH10	3.5	3.12331	3.450091	3.562446	3.691271	4.1	3.564591	4.367733	3.730518	3.359507

	HPM					PLA				
**Lactate**	Pre	Post 0	Post 30	Post 60	Next morning	Pre	Post 0	Post 30	Post 60	Next morning
MDH01	12	22.12196	13.14467	11.97104	6.431403	7.8	43.90855	18.55563	10.55945	10.11947
MDH02	7.4	15.82425	7.4	5.888079	14.71585	4.2	32.31565	7.642494	6.480715	12.36923
MDH03	9.4	25.18902	8.897959	9.001689	3.314225	11.2	24.67543	9.239095	5.923282	9.257285
MDH04	7.2	31.60937	19.0558	9.801681	9.567641	4.6	20.48611	11.87831	8.421802	4.525452
MDH05	6.4	21.11295	7.681245	6.346972	7.655463	4.4	18.59013	6.710204	7.205885	6.866801
MDH06	7	18.30644	5.903131	7.366661	10.59512	8.4	32.37807	12.31962	12.45606	10.01758
MDH07	7.4	24.6169	8.763462	6.701538	5.173248	5.8	29.21671	8.972963	9.07322	6.295753
MDH08	4.8	23.32621	9.77131	5.938432	4.518064	5	36.07767	9.226282	12.16304	7.09865
MDH09	18.4	20.77551	8.662974	6.190398	15.04822	9	21.61893	10.69779	8.229069	12.7692
MDH10	5.6	15.88426	11.63174	9.104029	8.380182	6.4	12.34566	6.076846	9.031782	6.143098

	HPM					PLA				
**UA**	Pre	Post 0	Post 30	Post 60	Next morning	Pre	Post 0	Post 30	Post 60	Next morning
MDH01	6.6	7.598412	8.763116	8.925777	8.298584	5.8	6.566681	7.920086	7.919586	7.809592
MDH02	5.7	5.511818	6.2	6.624089	7.150661	6	6.214547	6.713002	6.110388	7.784083
MDH03	5.1	5.357365	5.864564	5.674978	6.42131	5.5	5.55668	5.774434	5.732208	7.574142
MDH04	5.7	6.618211	7.576401	7.462643	7.231357	5.2	6.389232	6.69505	6.632169	6.582475
MDH05	6.4	6.351778	6.817105	7.020136	7.454003	7.1	7.455417	8.133581	8.132356	8.219352
MDH06	5.4	5.528181	6.208466	6.271617	7.063415	6.3	7.126695	8.149903	7.876624	8.127471
MDH07	5	5.063049	6.468269	6.396923	6.566046	4.5	4.839578	5.738523	6.119148	6.397297
MDH08	5.5	5.787037	7.276507	7.027145	6.777095	4.6	4.747062	5.692813	5.502326	6.084557
MDH09	6.5	7.15383	7.383216	7.157648	7.712213	6.5	7.20631	7.406162	7.406162	7.909279
MDH10	6.3	6.24662	6.801608	6.926979	7.981126	6.2	5.998946	6.5516	6.773836	7.390914

	HPM					PLA				
**CK**	Pre	Post 0	Post 30	Post 60	Next morning	Pre	Post 0	Post 30	Post 60	Next morning
MDH01	186.0	200.1	200.3	207.9	427.4	120.0	131.3	136.9	139.7	165.0
MDH02	118.0	117.3	128.0	135.6	175.1	147.0	146.3	149.8	143.5	152.5
MDH03	96.0	107.1	107.2	103.7	99.4	80.0	84.8	83.7	86.9	129.4
MDH04	131.0	148.2	155.0	168.2	538.5	150.0	176.5	181.4	217.9	311.6
MDH05	103.0	105.6	108.5	109.6	124.9	91.0	101.7	104.7	105.0	107.2
MDH06	195.0	204.8	204.6	198.1	147.5	269.0	297.4	303.3	279.3	279.7
MDH07	137.0	149.3	174.2	205.1	359.1	153.0	164.0	190.9	217.3	571.7
MDH08	105.0	109.5	113.3	111.8	103.1	80.0	97.5	96.2	93.6	88.2
MDH09	63.0	67.6	68.9	62.9	56.4	69.0	76.1	75.1	74.1	68.6
MDH10	94.0	108.0	109.4	109.8	110.7	135.0	136.5	136.7	135.5	111.3

Blood raw data (2)

	HPM					PLA				
**Mb**	Pre	Post 0	Post 30	Post 60	Next morning	Pre	Post 0	Post 30	Post 60	Next morning
MDH01	22.3	20.3	52.9	83.0	45.8	22.5	15.7	31.0	52.4	50.3
MDH02	26.6	27.6	39.6	52.6	42.6	24.3	24.5	57.6	42.1	46.0
MDH03	17.9	22.5	37.1	24.4	40.9	21.2	18.6	30.7	44.6	38.5
MDH04	17.5	24.2	77.6	128.3	68.6	21.8	32.7	77.5	95.4	48.4
MDH05	9.8	12.8	18.1	28.2	31.2	18.3	15.2	24.9	28.0	25.4
MDH06	42.5	45.9	61.9	57.1	65.4	65.8	63.4	124.6	124.7	54.3
MDH07	21.9	60.5	145.3	124.9	46.6	24.2	102.2	224.8	231.2	55.8
MDH08	17.2	19.5	48.2	55.8	32.1	25.5	22.0	39.8	39.1	34.7
MDH09	31.2	33.8	61.4	44.8	40.1	32.6	34.4	58.7	61.1	46.3
MDH10	18.3	42.1	47.6	46.0	33.9	21.9	38.4	46.9	47.1	40.1

	HPM					PLA				
**Leukocyte**	Pre	Post 0	Post 30	Post 60	Next morning	Pre	Post 0	Post 30	Post 60	Next morning
MDH01	8380	13004	21720	23281	8610	8100	11007	17402	19227	9064
MDH02	6200	6836		16171	5513	6120	8653	16576	17248	5332
MDH03	4510	5536	11537	12632	4723	4290	7553	11635	11866	4397
MDH04	5040	5334	15784	17030	5507	5400	10344	16252	15212	5760
MDH05	5900	5582	12751	13877	6668	5560	6158	16054	16079	6898
MDH06	4220	7540	15114	15490	5318	5460	8244	15305	15625	5302
MDH07	4990	6748	12165	12266	4387	4430	6318	12614	12523	4620
MDH08	4900	6134	12547	13114	4960	5380	6585	10080	11816	4634
MDH09	5580	8722	12207	13406	5577	5430	9979	15450	17631	5260
MDH10	4920	7014	17822	17080	5487	5460	12859	18439	16886	4675

	HPM					PLA				
**Urea nitoroger**	Pre	Post 0	Post 30	Post 60	Next morning	Pre	Post 0	Post 30	Post 60	Next morning
MDH01	14.1	12.9	14.8	14.6	13.7	14.4	13.9	16.4	15.9	14.8
MDH02	15.8	14.5	16.5	17.5	15.4	14.7	14.3	16.0	14.5	18.2
MDH03	14.7	13.6	14.8	14.0	16.6	14.9	13.9	14.9	14.2	17.8
MDH04	10.9	11.2	13.4	13.4	14.2	10.4	11.2	12.5	12.3	12.0
MDH05	11.6	11.1	12.6	12.8	11.5	14.0	14.6	16.0	16.3	14.9
MDH06	11.6	10.5	12.5	12.6	15.5	15.5	13.9	15.4	14.7	16.9
MDH07	11.3	10.5	12.6	12.4	11.6	11.3	10.9	12.8	13.7	13.0
MDH08	12.7	11.8	14.0	13.6	11.6	11.9	10.9	12.7	12.5	14.2
MDH09	12.1	12.5	13.5	13.5	13.8	9.7	11.0	11.7	12.2	12.5
MDH10	10.1	10.3	11.4	11.3	14.1	10.2	10.2	11.7	12.2	13.2

8-OHdG raw data

	HPM			PLA			
**8-OHdG concentration**	Pre	Post 0	Post 60	Pre	Post 0	Post 60	
MDH01	4.4	11.7	6.6	6.6	10.2	4.9	Outlier rejection by the statistical analysis
MDH02	8.3	10.9	6.7	5.9	7.8	7.7	
MDH03	10.9	8.5	5.1	4.9	5.4	4.2	
MDH04	3.7	7.8	5.4	8.2	10.5	6.7	
MDH05	8.8	12.6	7.7	9.3	14.6	6.3	
MDH06	7.6	9.0	6.6	5.9	15.4	5.5	
MDH07	7.5	8.4	5.9	4.6	14.8	8.3	
MDH08	8.2	11.9	4.8	8.1	9.1	5.1	
MDH09	10.3	8.2	6.4	7.9	8.7	7.3	
MDH10	9.9	9.0	8.2	8.3	14.0	9.9	

	HPM			PLA			
**8-OHdG production rate**	Pre	Post 0	Post 60	Pre	Post 0	Post 60	
MDH01	0.6	14.8	7.4	1.7	11.6	4.9	Outlier rejection by the statistical analysis
MDH02	10.4	12.6	7.4	7.1	8.9	9.5	
MDH03	15.9	9.7	6	5.9	7.2	4.9	
MDH04	3.4	9.8	7.7	5.4	14.1	7.6	
MDH05	5.4	14.1	7.1	16.1	16.7	6.3	
MDH06	14.2	11.9	8.0	7.1	21.3	7.7	
MDH07	9.2	9.7	7.5	2.4	16.0	9.3	
MDH08	10.5	14.6	3.0	10.6	10.4	4.2	
MDH09	11.1	10.0	6.2	9.8	9.1	10.0	
MDH10	11.5	7.5	9.9	4.2	21.2	10.0	

VAS raw data

	HPM					PLA				
**Fatigue**	Pre	Post 0	Post 30	Post 60	Next morning	Pre	Post 0	Post 30	Post 60	Next morning
MDH01	25	50	55	37	55	13	34	18	27	40
MDH02	1	71	47	39	14	23	69	48	41	63
MDH03	1	69	0	0	25	0	4	0	0	0
MDH04	1	87	51	48	45	21	64	52	43	39
MDH05	26	63	31	18	20	7	67	69	47	20
MDH06	25	49	64	44	29	16	74	86	69	59
MDH07	0	43	22	8	11	0	68	38	35	38
MDH08	3	36	55	50	42	11	71	67	57	20
MDH09	7	75	58	59	46	0	97	80	64	65
MDH10	5	67	31	4	15	4	66	26	8	32

	HPM					PLA				
**General muscle soreness**	Pre	Post 0	Post 30	Post 60	Next morning	Pre	Post 0	Post 30	Post 60	Next morning
MDH01	6	20	27	25	42	8	36	18	24	38
MDH02	0	46	31	26	6	1	47	34	23	17
MDH03	0	0	1	0	0	0	1	1	0	0
MDH04	2	53	66	52	53	2	49	39	36	32
MDH05	4	20	20	16	16	4	28	30	15	17
MDH06	11	34	33	17	7	11	55	55	38	34
MDH07	0	12	4	2	2	0	4	2	3	12
MDH08	3	11	11	3	6	8	29	32	14	17
MDH09	0	48	36	37	18	0	60	58	29	21
MDH10	4	38	23	3	14	4	55	26	9	33

	HPM					PLA				
**Lower muscle soreness**	Pre	Post 0	Post 30	Post 60	Next morning	Pre	Post 0	Post 30	Post 60	Next morning
MDH01	10	29	37	33	55	7	42	27	29	48
MDH02	4	75	51	35	29	1	73	46	46	25
MDH03	0	1	0	0	0	0	0	0	0	0
MDH04	2	59	70	56	79	2	55	47	44	49
MDH05	5	14	16	13	13	0	77	27	12	12
MDH06	19	54	34	16	12	11	73	70	49	52
MDH07	0	11	3	1	2	0	5	2	3	11
MDH08	2	25	11	6	6	8	28	30	14	17
MDH09	2	49	63	49	27	0	94	96	71	63
MDH10	7	40	20	5	13	5	59	25	9	32
